# Correlative VIS-fluorescence and soft X-ray cryo-microscopy/tomography of adherent cells

**DOI:** 10.1016/j.jsb.2011.12.012

**Published:** 2012-02

**Authors:** Christoph Hagen, Peter Guttmann, Barbara Klupp, Stephan Werner, Stefan Rehbein, Thomas C. Mettenleiter, Gerd Schneider, Kay Grünewald

**Affiliations:** a Oxford Particle Imaging Centre, Division of Structural Biology, Wellcome Trust Centre for Human Genetics, University of Oxford, Oxford OX3 7BN, UK; b Helmholtz-Zentrum Berlin für Materialien und Energie GmbH, Institute for Soft Matter and Functional Materials, 12489 Berlin, Germany; c Institute of Molecular Biology, Friedrich-Loeffler-Institut, 17493 Greifswald-Insel Riems, Germany

**Keywords:** DIC, differential interference contrast, GFP, green fluorescent protein, NEC, nuclear egress complex, PrV, pseudorabies virus, X-ray imaging, Live-cell imaging, Vitrification, Herpesvirus egress, Pseudorabies virus, Nucleoplasmic reticulum

## Abstract

Soft X-ray cryo-microscopy/tomography of vitreous samples is becoming a valuable tool in structural cell biology. Within the ‘water-window’ wavelength region (2.34–4.37 nm), it provides absorption contrast images with high signal to noise ratio and resolution of a few tens of nanometer. Soft X-rays with wavelengths close to the K-absorption edge of oxygen penetrate biological samples with thicknesses in the micrometer range. Here, we report on the application of a recently established extension of the transmission soft X-ray cryo-microscope (HZB TXM) at the beamline U41-XM of the BESSY II electron storage ring by an in-column epi-fluorescence and reflected light cryo-microscope. We demonstrate the new capability for correlative fluorescence and soft X-ray cryo-microscopy/tomography of this instrument along a typical life science experimental approach – the correlation of a fluorophore-tagged protein (pUL34-GFP of pseudorabies virus, PrV, the nuclear membrane-anchored component of the nuclear egress complex of the *Herpesviridae* which interacts with viral pUL31) in PrV pUL34-GFP/pUL31 coexpressing mammalian cells, with virus-induced vesicular structures in the nucleus, expanding the nucleoplasmic reticulum. Taken together, our results demonstrate new possibilities to study the role of specific proteins in substructures of adherent cells, especially of the nucleus *in toto*, accessible to electron microscopy in thinned samples only.

## 1 Introduction

Coming from an electron cryo-microscopy background (Grünewald et al., 2003, Vanhecke et al., 2011), soft X-ray cryo-microscopy, experienced hands-on at the HZB TXM at beamline U41-XM of the BESSY II electron storage ring in Berlin/Germany, offers several unique characteristics making it a complementary tool in structural cell biology. (i) The limit in sample thickness is in the micrometer range, and many mammalian cell models can be imaged intact with all their organelles and the nucleus well resolved to count and chart specific features (Schneider et al., 2010; for yeast cells, see Uchida et al., 2011). While for electron cryo-microscopy the cellular sample has to be as thin as possible, for X-ray cryo-microscopy one has to revisit the thickness limit of plunge freezing for cryo-immobilization (Dubochet et al., 1988). Tomography is key to mine the wealth of information, as soft X-ray cryo-microscopic images are too ‘crowded’ to reveal much detail, and the reconstructed volumes are almost cubes instead of thin slabs (Larabell and Nugent, 2010, Weiss et al., 2000). (ii) Radiation damage seems less of an issue (Schneider, 1998), at least at lower resolution. Thus, a ‘low dose approach’ as in electron cryo-microscopy is not necessary, and the entire dynamic range of the detector is used yielding high signal/low noise data. (iii) Imaging in the wavelength region between the innershell absorption edges of oxygen and carbon (2.34–4.37 nm), known as ‘water window’ (Thieme et al., 1993, Wolter, 1952), ice contamination is accordingly hard to see, and is mostly not interfering with data acquisition. (iv) One key part of the instrument, the zone plate focusing element, does not look like an objective, but is a high-precision in-house development of the HZB Nano-Lab (Rehbein et al., 2009, Werner et al., 2010). (v) Fascinatingly, the object is illuminated with soft X-ray radiation by an elliptically shaped capillary condenser mirror of a defined length (Guttmann et al., 2009). (vi) Even though it seems conceptionally to be a ‘light’ microscope, one can see nuclear pores with it (Schneider et al., 2010). Taken together, it would be nice to have such an instrument in our own lab. Unfortunately, it (still) needs a ‘huge lamp’ (for development of a laboratory soft X-ray microscope, see Bertilson et al., 2011b).

But even good things can be improved. As a cell biologist, reading about the plan to establish correlative fluorescence and X-ray cryo-microscopy was stimulating (Le Gros et al., 2009, McDermott et al., 2009, Schneider et al., 2006), getting hands on it is thrilling. Since beginning of 2011 we have worked with the newest extension of the HZB TXM in Berlin – an in-column epi-fluorescence and reflected light microscope (for technical description see Schneider et al., this issue). The goal is to correlate results of cell imaging using a rainbow of fluorescent tags to elucidate the role of specific proteins in the cell (Wiedenmann et al., 2009, Wombacher and Cornish, 2011) with nanometer-resolved structures from soft X-ray microscopy (Falcone et al., 2011), in vitreous samples, i.e. with the best possible preservation of ultrastructure (Dubochet, 2007). In electron cryo-microscopy/tomography, already several correlative approaches and solutions exist (for a recent review, see Briegel et al., 2010).

Here, we have chosen an application of one of our recent projects – characterization of the herpesvirus ‘life’ cycle with cryo-microscopy (Ibiricu et al., 2011, Maurer et al., 2008) – to demonstrate as a proof of principle the new capability of the HZB TXM instrument. Herpesviruses are complex viruses with a distinct replication cycle involving cytoplasmic and nuclear compartments of infected cells. Viral capsids assemble in the nucleus and gain access to the cytosol by vesicular transport through the nuclear envelope, mediated by the viral nuclear egress complex (NEC) consisting of pUL34, a type II membrane anchored protein, interacting with pUL31 (for review see Mettenleiter et al., 2009). Heterologous coexpression of just these two proteins in mammalian cells results in the budding of vesicles from the inner nuclear envelope into the perinuclear space (Klupp et al., 2007).

We share our experience in sample preparation for soft X-ray cryo-microscopy, and discuss further improvements.

## 2 Materials and methods

### 2.1 Cells

Porcine epithelial-like embryonic EFN-R kidney cells stably coexpressing pseudorabies virus (PrV) pUL31 and pUL34, the latter fused to green fluorescent protein (GFP; pUL34-GFP; cell line designated as BK/EFN/UL31/34gfp, catalog No. RIE 1083 of the Collection of Cell Lines in Veterinary at the FLI, Greifswald-Insel Riems, Germany), were grown in Dulbecco’s Modified Eagle Medium (Gibco-Invitrogen, Karlsruhe, Germany) supplemented with 10% (w/v) fetal calf serum and 1% (v/v) PSN Antibiotic Mixture (Gibco-Invitrogen). Generation and characterization of the cellular model are given in Klupp et al. (2007).

### 2.2 Sample preparation for X-ray cryo-microscopy

**Fig.1:**
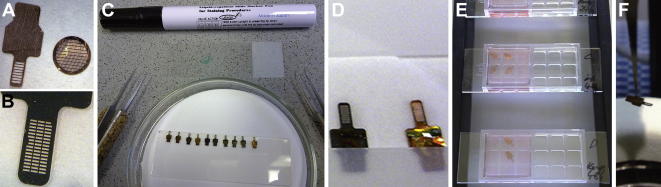
Sample grid preparation for soft X-ray cryo-microscopy/tomography at the BESSY II U41-XM beamline. (A) Due to the confined space for sample tilting in the microscope, a special grid design (IFR-1, left) is needed. For comparison, a standard 400 × 100 mesh grid for electron microscopy is shown (diameter: 3.05 mm, right). (B) Retaining the outer dimensions, an improved mesh design (HZB-2) with smaller slots helps to stabilize standard perforated carbon film (tested: Quantifoil R2/2). Furthermore, it alleviated targeting for correlative microscopy. (C) In order to prevent liquid to spread over the whole grid, liquid blocker (PAP pen) was applied as a thin line near the observation area of the grid. (D) Alignment marker bead suspension was blotted and dried on the carbon film of the glow-discharged grid. Note the green liquid blocker line on the carbon-coated side of the right grid. (E) On-grid cell incubation was performed in commercially available microscope slide growth chambers. (F) Grid handling with tweezers, and finally vitrification by plunge freezing of the sample, has to spare the clamping end of the grid for proper mounting in the Gatan model 630 specimen cryo-holder.

A detailed “*Protocol for partially coherent X-ray microscopy*” is provided in the Supplement of Schneider et al. (2010). Here, we highlight improvements or alternative procedures along this description, in parts depicted in Fig. 1.

For on-grid cell cultivation, custom-designed gold grids (new design with smaller slots, specified as HZB-2) were purchased from Gilder Grids, Grantham, UK. These grids were coated with a R2/2-perforated carbon foil by Quantifoil Micro Tools GmbH, Jena, Germany. This coat is a standard grid support in electron cryo-microscopy, and is compatible with adherent cell growth (Ermantraut et al., 1998, Maurer et al., 2008). Before cell incubation, grids on a glass slide with the carbon coat upwards were treated in a PDC-002 plasma cleaner (Harrick Plasma, Ithaca, NY, USA) to increase hydrophilicity. The coated side of the grid was marked with a permanent marker pen, and the mesh area was demarcated from the rest of the metal area of the grid by applying a thin line of PAP pen liquid blocker using the 45°-bended tip of tweezers (both items from Plano GmbH, Wetzlar, Germany). These grids, blocked from liquid spreading by the PAP pen line only on the carbon coat side, were placed on Whatman No. 50 hardened filter paper carbon-side up, and 2 μl of a 1:4 dilution of 200-nm FluoSpheres (carboxylate-modified polystyrene microspheres, red fluorescent proprietary dye, excitation max.: 580 nm, emission max.: 605 nm, 2% solids, catalog No. F-8810, Invitrogen, UK) were applied as alignment and correlation markers to the carbon-coated mesh area of the grids and thereby sucked through to the filter paper underneath. After 5 min of drying, the grids were submerged in 1 ml of fresh complete medium in plastic microscope slide growth chambers (μ-slide 2×9 well, Ibidi GmbH, Munich, Germany). At 90% confluency, cells from 25-ml cell-culture flasks were trypsinized, and were seeded onto the grids in the growth chamber slides at a cell density of approximately 3 × 10^4^ cm^−2^. The slides were incubated on an aluminum rack at 37 °C, 5% CO_2_ and approximately 90% humidity. Medium was exchanged after 6 h of incubation to remove non-attached cells and cell debris, which was repeated at least every 24 h.

Live-cell light microscopy was applied by default before cryo-immobilization to characterize the status of the cell material (for technical details, see Hagen and Grünewald, 2008). First, the grids were transferred to a fresh microscope slide growth chamber with medium, and then a three channel grid scan was performed (objective 20×, N.A. 0.4; phase contrast; GFP: eGFP HQ filter set F41-017 from AHF analysentechnik AG, Tübingen, Germany; FluoSpheres: TRITC/rhodamine filter set F11-007 from AHF).

Plunge freezing was performed essentially as described for standard sample grids in electron microscopy (Maurer et al., 2008), and for IFR-1 grids as detailed in Schneider et al. (2010). The grid was taken from the incubation chamber, and the area to be later clamped into the cryo-transfer holder was dried by applying a filter paper to the non-coated grid side. There, a second fine liquid blocking line was applied, and the tweezers with the grid were clamped in a home-made plunge freezer (Rigort et al., 2010). Two microliter of a 1:10 dilution in culture medium of a 200-nm red FluoSpheres suspension were added to the mesh area of the grid, and blotted manually with a bent strip of Whatman No. 1 filter paper from the non-coated grid side for 2 to 3 s immediately before vitrification by the gravity-driven plunging apparatus in a ethane/propane mixture cooled by liquid nitrogen (Tivol et al., 2008). It was important not to pinch the clamping area of the HZB-2 grid with the tweezers, especially during plunge freezing, to assure later smooth transfer to the Gatan model 630 cryo-holder of the soft X-ray cryo-microscope.

### 2.3 In-column light cryo-microscopy

A detailed technical description of the instrument is provided by Schneider et al. (this issue). HZB-2 grids were cryo-assembled into the Gatan model 630 cryo-holder and transferred to the tilting stage of the soft X-ray cryo-microscope in a way that the vitreous cells faced the X-ray beam. Next, the in-column light cryo-microscope was activated. Parking of the capillary condenser, bringing the light microscope with its 100× objective (N.A. 0.75) in front of the sample grid and positioning filter sets took less than five minutes. Thus, frequent changes between the light and the X-ray microscope working positions were possible. Differential interference contrast (DIC) was used first to bring the grid in register with its positioning mask in the in-house microscopy software, and then to get a quick overview on the intactness of the holey carbon support film in all 48 grid slots. Regions of interest were selected with the GFP channel. In order to relocate these spots later on, we found it useful to scan the whole grid slot (i.e. a montage of four image frames), here with three channels: the green emission filter set for GFP detection, the DIC channel and the red emission filter set for detection of the FluoSpheres (for filter details, see Schneider et al., this issue). Image stitching was done with AxioVision 4.8.2 (Carl Zeiss MicroImaging GmbH, Göttingen, Germany).

### 2.4 Soft X-ray cryo-microscopy/tomography

After switching back to the X-ray cryo-microscope working position, a target structure, here the nucleus, suitable for tomography was chosen from the grid slot scanned with the light cryo-microscope before. As fast changes between zone plate objectives with different numerical apertures are not yet implemented in the HBZ TXM, and screening has to be done in the final magnification, such a light microscopic map of the corresponding region was very helpful to find objects of interest efficiently. At extreme tilt angles, the sample position was checked for obstruction by grid bars or by contamination, and for stability of the focus position. Exposure time was left at 4 s or longer to exceed condenser wobbler frequency, in order to obtain a more stable flatfield, and the monochromator slit was adjusted to exploit the full dynamic range of the camera. Tilt series acquisition was started after taking a control exposure at a tilt angle of 0°, and was run either script-controlled in an automatic fashion, or with user interventions correcting focus and/or sample position if necessary. The tilt series presented here in detail was taken with the 25 nm zone plate objective (object pixelsize 9.9 nm), from −60° to 60° tilt angle with 1° spacing, by default. After finishing tilt series acquisition, a second control image at 0° tilt angle was obtained, and 10 empty images for flatfield correction were recorded (Schneider et al., 2010). Tilt series presented in Fig. 5 as slices from tomographic reconstructions were taken with the 40 nm zone plate objective, either with an object pixelsize of 15.5 nm (Fig. 5A, B), or 10 nm by changing the camera position along the beam axis (post-zone plate magnification; Fig. 5C).

Tomographic reconstruction was performed with the Etomo GUI of IMOD (Kremer et al., 1996, Mastronarde, 2008). If not stated otherwise, tomograms and slices presented here are binned with a kernel of 2 × 2 × 2, and slices are shown in the corresponding voxelsize thickness. Visualization was performed with Amira 5.2 (Visage Imaging GmbH, Berlin) and Adobe Photoshop CS4 (Adobe Systems Inc.).

Resolution assessment of the soft X-ray tomographic reconstructions was done based on Fourier ring correlation according to the so-called noise-compensated leave-one-out (NLOO) criterion using the software ELECTRA (Cardone et al., 2005). Calculations where performed in unfiltered and unbinned reconstructed full volumes providing a rather conservative measure of resolution, as compared to published results of soft X-ray subtomograms (Carrascosa et al., 2009).

## 3 Results

Based on the detailed description of the HZB full-field transmission X-ray microscope (TXM) at the beamline U41-XM of the BESSY II electron storage ring (Schneider et al., this issue), and of the sample preparation steps (Schneider et al., 2010) we present here latest developments, for instance a new grid design (Fig. 1), and especially the application of the recently established in-column epi-fluorescence and reflected light cryo-microscope. We demonstrate the potential of a combined approach by the localization of a fluorescently tagged viral protein in subcompartments of the nucleus of adherent mammalian cells, in samples featuring the best possible ultrastructural preservation by vitrification.

A special grid design is necessary to reach higher tilt angles for tomography in the already space-augmented object plane of the soft X-ray cryo-microscope (Fig. 1A). A cellulose nitrate film was earlier proposed as support for the sample (Schneider et al., 2010). However, this film exhibited auto-fluorescence and was suboptimal for blotting of the sample before plunge freezing. Furthermore, home-made holey carbon coatings were difficult to reproduce on these grids. Thus, we tested standard perforated carbon support foil (Quantifoil). With the relatively large slot size of the IFR-1 grid design (Fig. 1A) the coat was prone to damage, especially during plunge freezing. Without changing the outer shape and dimensions of the grid, a new design of the mesh with smaller slots was established (Fig. 1B). Besides stabilizing the carbon coat, the new design helped us in the sense of a finder grid to relocate a region of interest faster and more reliably, by providing a higher density of landmarks like grid bars or mesh corners. Furthermore, the larger area of grid metal in contact with the vitreous sample should also yield improved heat-dissipation of absorbed X-ray energy. Additionally, the new grid design is less fragile.

A problem with the overall grid design was that all liquid brought to the hydrophilized mesh did not form a drop but spread immediately up to the clamping area. In order to achieve reproducible results for the blotting shortly before plunge freezing, a thin line of liquid blocker was applied (PAP pen; Fig. 1C, D). It improved blotting of alignment marker beads on the carbon-coated surface (Fig. 1D), and was compatible with cell growth on the grids (Fig. 1E). The latter step was done in commercially available microscope slide growth chambers to allow for microscopic control of the cultivation status, and for grid pre-scanning before plunge freezing (Fig. 1F).

**Fig.2:**
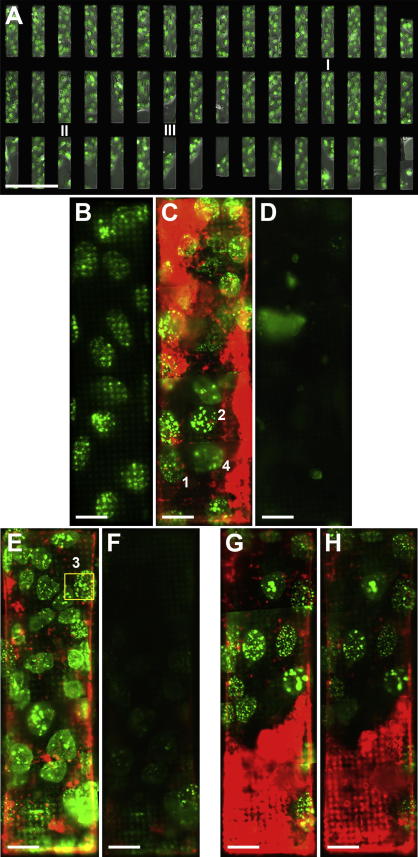
Correlative live-cell pre-scanning and in-column fluorescence cryo-microscopy of PrV pUL31/pUL34-GFP coexpressing EFN-R cells grown on a HZB-2 grid. For most images, two or three detection channels were combined: gray, phase-contrast image at the focal plane of the cell nuclei; green, GFP fluorescence from tagged PrV pUL34; red (only in light cryo-microscopic images), red fluorescence of FluoSpheres beads (diameter 200 nm, 580/605 nm excitation/emission). (A) Montage of live-cell light microscopic images taken at 20×-magnification after 36 h of incubation at 37 °C and 5% CO_2_, exhibiting optimal cell density for our experiments. Higher resolved imaging data were acquired from grid slots marked below with Roman numerals. (B) Enlarged view of grid slot number I illustrating the confinement of the target protein, GFP-tagged PrV pUL34, to the nuclei. Note the shading/absorption of epi-fluorescence by the carbon support foil. (C) Montage of fluorescence cryo-microscopic images taken with a 100× long distance objective (N.A. 0.75) of the same grid slot number I shown in (B), after further 3.5 h of incubation and plunge freezing. The images were gathered with the light cryo-microscope installed inside of the HZB TXM. The Arabic numerals mark the nuclei (and the order) where subsequently soft X-ray tilt series had been taken (tilt series 3 was acquired from grid slot II, *cf*. E). Red fluorescing FluoSpheres were applied, in excess as correlation and alignment markers, before cell seeding and incubation, as depicted in Fig. 1D, and additionally before plunge freezing. (D) Montage of in-column fluorescence cryo-microscopic images taken of the same grid slot number I shown in (B) and (C), after X-ray exposure for the four tilt series marked in (C) and in (E). As camera settings were kept exactly the same as in (C), it demonstrates the almost complete bleaching of both fluorophores. Another two grid slots of the sample grid depicted in (A) (E, F – II; G, H – III) were imaged by in-column fluorescence cryo-microscopy before (E, G) and after (F, H) X-ray exposure for four tilt series taken close to the spots marked by Arabic numerals (E – for 3, camera frame/X-ray irradiated area at 0° tilt angle marked by yellow rectangle; *cf*. C for 1, 2 and 4). Note the fluorescence intensity gradient in (F) where highest bleaching was observed near the exposure spot 3. More distant areas were also affected, due to horizontal tilting during tilt series acquisition, stray light and/or inefficiency of the condenser beam stop. As a control (G, H), grid slot number III was not exposed to direct X-rays, neither for searching nor for tilt series acquisition. Scale bar is 200 μm (A) or 20 μm (B–H), respectively.

Fluorescence microscopy is our main tool to characterize samples before cryo-immobilization and (ultra)structural analysis. Already the live-cell pre-scanning overview of the sample grid, obtained at low magnification, provided important information for the following experimental steps (Fig. 2A). (i) The carbon coat was intact in almost all grid slots. (ii) Cell growth had reached a level of confluency optimal for further analysis, forming a flat monolayer with a high target density, in our case, the nuclei. (iii) The GFP-tagged target protein PrV pUL34-GFP reached its final cellular localization, the nucleus (Fig. 2B), forming fluorescent ‘speckles’ within the nucleoplasm (Fig. 2C). These ‘speckles’ were described in Klupp et al. (2007) as perinuclear vesicles containing the NEC (for review, see Mettenleiter et al., 2009). The enlarged view of the GFP channel image of a grid slot in Fig. 2B also shows that the Quantifoil carbon support film being located between the objective lens and the cells growing on the support film surface in this standard epi-fluorescence setup of an inverted light microscope perturbed the observation of the nuclei to some extent. To avoid this shading effect at the higher magnification of the light cryo-microscope in-column of the soft X-ray cryo-microscope, we oriented our sample grids with the cell layer towards the light objective and, consequently, the X-ray source. Comparison of the same grid slot before (Fig. 2B) and after 3.5 h of incubation and, finally, plunge freezing (Fig. 2C) suggested that cells were moving and dividing during that period. Exposure to X-rays under cryo-conditions resulted in strong bleaching of both fluorophores in the sample, surprisingly even more of the proprietary dye in the FluoSpheres polystyrene beads than the live-tag GFP (Fig. 2D).

For practical reasons, the observation of bleaching of the fluorophores by soft X-ray exposure is important. It means that, for a certain sample area and the fluorophores used in this study, in-column fluorescence can only be observed before X-ray analysis. To study this issue further, we took a tilt series in the upper corner of another grid slot and treated a further grid slot as control (Fig. 2E–H). From this experiment it became clear that also more distant areas of the X-ray irradiated spot were bleached (Fig. 2F). This was supported by the observation that searching with higher binning and a reduced monochromator slit, thus applying only approximately 1/100th of the dose typically used for a full tilt series, already resulted in reducing fluorescence intensity by more than 80%. The slight reduction of fluorescence intensity in Fig. 2H might had been elicited by excitation with blue and yellow light during the first screening but, more likely it was induced by X-ray stray light, since strongly increased stability against photobleaching under cryo-conditions has been reported (Le Gros et al., 2009, Sartori et al., 2007, Schwartz et al., 2007).

The reflected brightfield or DIC channel of the in-column light cryo-microscope was used to align the grid to the grid mask of the microscope in-house software. Additionally, its application helped to avoid sample areas with heavy ice contamination, ice cracks or damage of the carbon coat caused during plunge freezing. Using the Quantifoil perforated carbon coat we could not recognize the cell shape or intracellular features because of strong light reflection of the carbon film and the large contrast between the carbon surface and the perforated holes. Nevertheless, together with the fluorescence channels, the search for interesting sample spots suitable for X-ray cryo-tomography was sped up, especially owing to the wider field of view as compared to the X-ray microscope. To some extent, blurring of features in the fluorescence channels was used as guidance to sort out regions with excessive sample thickness. However, sample spots too thick for X-ray cryo-tomography and/or showing plunge freezing artifacts remained the main problem hindering correlation of fluorescence data with soft X-ray results.

**Fig.3:**
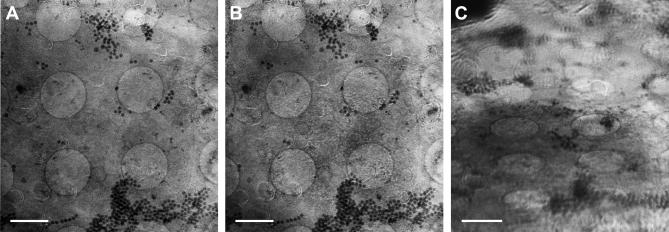
Soft X-ray tilt series. Data of nucleus 2 are presented (*cf*. Fig. 2C). (A) Shows the tilted area before, and (B) after series acquisition, both at a tilt angle of 0°. (C) Depicts the first image of the series, taken at −60°. Images (A) and (B) were aligned to each other, and comparison by dynamic superimposition revealed small geometric changes, especially at the surface of the sample (*cf*. Supplement Movie 1 – dynamic superimposition A/B, Movie 2 – pre-aligned tilt series). Images obtained at high tilts (C) illustrate the depth of focus limitation of the optical setup, as details near the horizontal tilt axis remained focused whereas peripheral features exhibited blurring (see, for instance, the large horsehead nebula-like aggregate of FluoSpheres near the right bottom corner of the image). However, the contrast of FluoSpheres beads was sufficient for precise marker-based alignment, even at high tilts (C), in this thin sample (sample thickness: 3.4 μm). For sample details, see Fig. 2. Scale bar is 2 μm.

**Fig.4:**
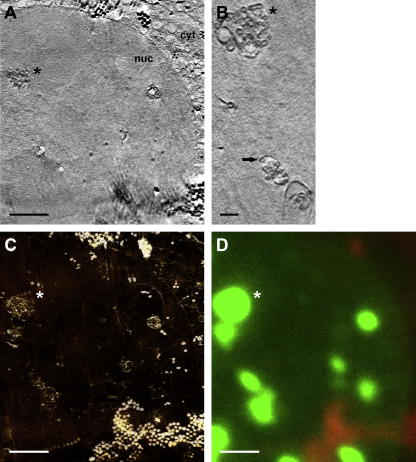
Fluorescent ‘speckles’ correlate with perinuclear vesicles in soft X-ray cryo-tomograms. (A) Slice of a tomographic reconstruction of the tilt series shown in Fig. 3 (*cf*. Fig. 2C, nucleus 2) exhibiting cross sections of perinuclear vesicles (an asterisk marks the largest conglomerate in A–D). (B) A different slice of an unbinned and Gaussian-filtered subtomogram of the series reveals the perinuclear vesicles more clearly (arrow; *cf*. Supplement Movie 3, complete subtomogram). (C) Negative contrast volume-rendering visualizes the extension of the conglomerates of the perinuclear vesicles in the entire volume of the nucleus (*cf*. Supplement Movie 4, animated volume). (D) Enlargement of the in-column fluorescence cryo-microscopic image of nucleus 2 allowed recognition of the large FluoSpheres aggregate near the right bottom corner of the soft X-ray reconstruction shown in (C) (*cf*. Fig. 3, tilt series), thus allowing for alignment of both channels. (C) and (D) cover the same area of the sample and show perfect correlation of the green fluorescent ‘speckles’ (D) with the aggregations of perinuclear vesicles in (C) (*cf*. Supplement Movie 5, dynamic superimposition of C and D). For sample and channel details, see Fig. 2. Scale bar is 2 μm (A, C, D), or 500 nm (B), respectively. cyt, cytoplasm; nuc, nucleus.

Prior searching for regions of interest with the in-column light cryo-microscope was also useful to minimize radiation damage. At least with the 25 nm zone plate objective, in almost all tilt series taken we noticed slight structural differences between the control images before and after tilt series acquisition (Fig. 3A, B; Supplement Movie 1). Fortunately, effects like increase of graininess were mostly confined to the X-ray-exposed surface of the sample (Fig. 3A, B; Supplement Movies 1 and 2), and did not disturb subsequent marker-based alignment and tomographic reconstruction (Fig. 4; residual error of this particular tilt series alignment with seven marker points: 0.64 with a standard deviation of 0.39). In this study, we used FluoSpheres as alignment markers in X-ray cryo-tomography (Fig. 3), and as correlation markers (Fig. 4C, D; Supplement Movies 4 and 5). These polystyrene beads exhibited sufficient X-ray contrast to locate them also in high tilt images (Fig. 3C), in thinner samples (up to 5 μm tested). Due to high particle concentration, a subset of the FluoSpheres formed aggregates which were large enough to recognize them in the fluorescence channel, thus serving as landmarks to align the red fluorescence channel to the soft X-ray channel. After correction of a small offset between the red and green fluorescence channel by using light reflected from the perforated carbon foil, it was possible to correlate the green fluorescing ‘speckles’ in the nucleus with aggregations of perinuclear vesicles found in the X-ray tilt series reconstruction (Fig. 4; Supplement Movies 3–5). For some fluorescence spots in the periphery of the region of interest, a complementary structure in the tomographic reconstruction was not obvious. In these areas, reconstruction quality suffered from missing image information due to the relatively large tracking error of the tilt series (Supplement Movie 2). We observed the correlation of GFP-tagged PrV pUL34 with perinuclear vesicles in tomographic reconstructions in three more nuclei (not presented).

**Fig.5:**
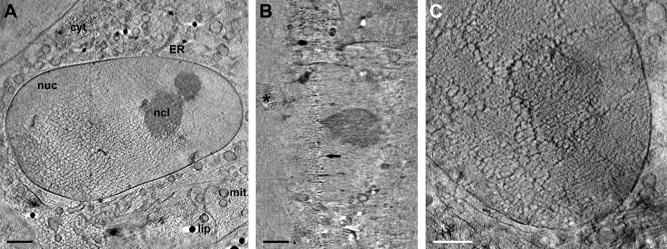
Typical sample-preparation related artifacts in X-ray cryo-tomography. Sometimes, especially in thicker samples (>8 μm), X-ray tomograms showed a central lattice bearing features typical for water crystallization, i.e. a segregation and aggregation pattern (A, X/Y-slice, the thin line marks the Z/Y-slice shown in B where the lattice is highlighted by an arrow; asterisk, ice contamination on the cell surface). It demonstrates the thickness limit of the vitrification method. A similar but clearly discernable pattern of artificial structures was observed in overblotted/dried samples (C). For sample details, see Fig. 2. Scale bar is 2 μm. Tilt series of the tomographic slices presented in this figure were acquired with the 40 nm zone plate objective. cyt, cytoplasm; ER, endoplasmic reticulum; lip, lipid body; mit, mitochondrium; nuc, nucleus; ncl, nucleolus.

Schneider et al. (2010) pointed out that the principal limitation for X-ray cryo-microscopy was poor cryo-preservation of the sample. Although this was no longer a major obstacle using Quantifoil perforated carbon coat, we observed occasionally the inherent vitrification limit of plunge freezing in thicker samples (>8 μm). In these samples, a central layer exhibiting features typical for water crystallization and thus segregation of cell material appeared (Fig. 5A, B). That matches the theoretical model, i.e. the freezing rate dropping hyperbolically along the sample thickness from both sides, leaving a central region where the freezing rate is too low to achieve formation of vitreous/amorphous water (Studer et al., 1995). Normally, any badly frozen samples exhibiting crystalline-ice caused artifacts throughout the entire sample were recognized already before tilt series acquisition and could be avoided, as well as overblotted/dry sample regions (Fig. 5C). For X-ray cryo-microscopy of samples generally too thick for plunge freezing (>15 μm), high-pressure freezing can be applied (Elbaum et al., this issue).

**Fig.6:**
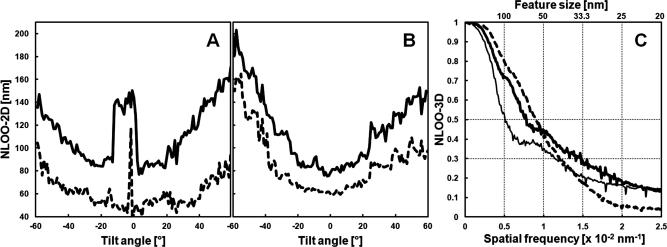
Resolution estimated according to Cardone et al. (2005) by Fourier ring correlation analysis in soft X-ray cryo-tomograms calculated from tilt series obtained under different imaging conditions in vitreous adherent cells/nuclei. (A) Variation of resolution with tilt angle, as measured by NLOO-2D, for a tomogram calculated from a tilt series of a 4.4 μm thick sample, measured with the 25 nm zone plate objective (object pixel size: 9.9 nm), and plotted at two cut-off thresholds (solid line: 0.5, dashed line: 0.3). The image taken at a tilt angle of −2° in this tilt series was blurred deteriorating resolution at both cut-off thresholds. The broad appearance of the outlier-like resolution measurement in the 0.5 threshold data (−12° to 0°) was caused, additionally, by oscillations in the individual NLOO-2D curves. (B) Tilt-angle dependent variation of NLOO-2D for a tomogram calculated from a tilt series of a 4.5 μm thick sample, measured with the 40 nm zone plate objective and post-zone plate magnification (object pixel size: 10 nm), and plotted at two cut-off thresholds (solid line: 0.5, dashed line: 0.3). (C) Resolution curves, depicted by NLOO-3D as a function of the spatial frequency, in tomograms presented in (A) (here: thick solid line) and (B) (here: dashed line). Additionally, data of a tomogram calculated from a tilt series of a 7.4 μm thick sample, measured with the 25 nm zone plate objective (object pixel size: 9.8 nm), are given (thin solid line). Cut-off thresholds at 0.3 and 0.5 are marked (dotted horizontal lines).

When tomographic data of larger objects, for instance of the complete nucleus, are needed (Fig. 5A, B), working with the 40 nm instead of the 25 nm zone plate objective is advisable to keep the object of interest in the field of view. An increase in magnification can still be achieved within minutes by changing the camera position and offers, to a certain extent, the possibility to search more efficiently at lower magnification before the actual tilt series acquisition at high magnification. Using the 40 nm zone plate might save X-ray dose because the practical diffraction efficiency of the 1st order of the 40 nm zone plate is supposed to be higher. This consideration was supported by the observation of less severe or not detectable X-ray-induced changes of the irradiated cell surface under comparable imaging conditions. Furthermore, an increase of the post-zone plate magnification to the same object pixelsize as for the non-magnified 25 nm zone plate image (∼10 nm; Fig. 5C) resulted in a similar Rayleigh resolution, at least in the periphery of tomographic slices where we measured the smallest resolvable distance between inner and outer nuclear membrane, as reported for the 25 nm zone plate objective (Schneider et al., 2010). This position-dependent lack of improvement in three-dimensional resolution by the objective with the higher numerical aperture was very likely caused by the decreased depth of focus of the 25 nm as compared to the 40 nm zone plate objective, thus deteriorating image information from points distant of the focus plane, especially at higher tilt angles and far away from the tilt axis (*cf*. Fig. 3C and Supplement Movie 2; for theoretical considerations, see Bertilson et al., 2011a and Knöchel 2005; for a detailed study on a similar problem in scanning transmission electron microscopy/tomography, see Biskupek et al., 2010). The highly anisotropic nature of spatial resolution in our soft X-ray tomograms of specimens with flat sample geometry and a limited tilt range during data acquisition was also observed performing resolution assessments based on Fourier ring correlation analysis (Fig. 6), a more appropriate approach than using the Rayleigh criterion, at least in weighted-backprojection reconstructions calculated from tilt series obtained with a partially incoherent microscope design (Schneider et al., 2010). Thus, resolution was optimal only in a narrow angular range around the untilted position (Fig. 6A, B), likely reflecting in part, again, the depth of focus limitation of zone plate objectives (*cf*. Supplement of Schneider et al., 2010). Also according to this method for resolution assessment, the 25 nm zone plate objective was seemingly inefficient in gaining resolution as compared to the 40 nm objective used at a comparable object pixelsize and tomogram/sample thickness, at least for a cut-off threshold of 0.5 (Fig. 6A–C). With a cut-off threshold of 0.3 or 0.25, as employed for the FSC_e/o_ criterion resolution assessment in purified vaccinia virus particles (Carrascosa et al., 2009), superiority of the 25 nm objective was demonstrated, supporting theoretical considerations to use that threshold for a more reasonable resolution estimate (Cardone et al., 2005). However, resolution in tomograms is influenced by many factors, for instance, by errors in the alignment of the tilt series, rendering comparison and causal analysis difficult. For example, tomographic reconstruction of a tilt series of a thicker sample, obtained with the same zone plate objective, exhibited reduced resolution (Fig. 6C).

## 4 Discussion

“*Perhaps the most exciting recent development in soft X-ray tomography imaging is the correlated use of X-ray and cryo-light microscopy* (Le Gros et al., 2009)*. Using this combination of modalities, it is possible to determine the location of labeled proteins using fluorescence, and then place this information directly into a high-resolution tomographic reconstruction of the cell (…). This represents an enormous leap forward in our ability to image cells, and cellular processes.*” (Quoted from McDermott et al., 2009) Here, we describe our approach, using the HZB TXM in Berlin, towards this combination of light and X-ray cryo-microscopic modalities correlating specific and structural information. We provide a proof of principle with a biological sample for which the cellular localization of the fluorescence-tagged protein has already been shown non-correlatively by confocal laser-scanning microscopy and immunolabeling in chemically fixed electron microscopic samples (Klupp et al., 2007). With the extraordinary capability of soft X-ray cryo-tomography to deliver nanometer-resolved structural data from large volumes, in this report the whole nucleus, we studied the composition of vesicles from the nuclear envelope driven by the herpesvirus NEC consisting of pUL34 and pUL31 in more detail. We found, that the fluorescent ‘speckles’/perinuclear vesicles were not only located near the nuclear envelope but also in the center of thicker nuclei. Thus, the NEC-driven vesicle formation occurs throughout the nucleus, resulting in vesicles embedded in nucleoplasmic reticulum (Malhas et al., 2011). A detailed description of our biological findings goes beyond the scope of this paper and will be done elsewhere.

Our results open the door for many improvements. Because of the overall design of the instrument allowing for tilting of flat specimen like adherent cells, and due to the confined space near the object plane, the in-column light cryo-microscope of the HZB TXM at the beamline U41-XM of the BESSY II electron storage ring will not achieve the high resolution of the external light cryo-microscope coupled to the transmission soft X-ray cryo-microscope XM-2 in the beamline 2.1.2 at the Advanced Light Source, Lawrence Berkeley National Laboratory, Berkeley, CA (McDermott et al., 2009). There, a higher numerical aperture objective (N.A. 1.3) will be applied, immersed in liquid propane or isopentane as refractive index matching fluid in contact to the vitreous sample in a glass capillary (Le Gros et al., 2009). Nonetheless, the relatively open concept of the in-column light cryo-microscope of the HZB TXM allows for changes, for instance, of the light source, to establish laser scanning excitation in order to implement other, even three-dimensional imaging modalities. The power of such an approach has already been demonstrated in-column of an electron microscope (Agronskaia et al., 2008). Another improvement would be to establish a fiberoptic-based illumination for transmission light microscopy, in order to get more detailed information from the brightfield/DIC channel.

Due to their performance in similar correlation approaches in electron microscopy (Kukulski et al., 2011), we applied FluoSpheres as alignment and correlation markers in soft X-ray cryo-microscopy. This was feasible in relatively thin samples, since the contrast of these polystyrene beads was as high as that of lipid bodies. However, at higher tilt angles in samples with optimal thickness for soft X-ray cryo-tomography of nuclei (5–8 μm) this contrast is not sufficient, in particular when coming together with blurring due to the limited depth of focus of high-resolving zone plate objectives. Thus, different spherical particles showing a contrast similar to gold beads, but also providing a bright and, preferably, more soft X-ray radiation-resistant fluorescence signal than the fluorophores in this study, should be deployed. Good candidates for this are quantum dots in the form of size-tunable photoluminescent aqueous CdSe/ZnS microspheres (Zhou et al., 2011). Multiwaveband microspheres (TetraSpeck) might be applied to fine align all detection channels of the HZB TXM, and might be used in calibration specimen to characterize the correlation accuracy between the fluorescence channels and X-ray detection in more detail (Agronskaia et al., 2008, Kukulski et al., 2011).

It is not surprising that soft X-ray irradiation, inducing in the sample most likely “*liberation of energetic primary electrons through photoelectric absorption and the Auger effect*” (quoted from Jacobsen et al., 1993), causes bleaching of fluorophores. Similar effects were observed in correlative light and electron cryo-microscopy (Lučić et al., 2007). As we did not detect structural changes within the present spatial resolution in our bleached samples after X-ray exposure, our results underline that there are other mechanisms for radiation damage, more dose-sensitive than observable mass loss (Beetz and Jacobsen, 2002), and that we have to expect radiation-induced artifacts in our samples after ∼10^9^ Gy total exposure during a standard tilt series (Schneider et al., 2010). Thus, a critical soft X-ray dose for C=O bond breaking of only 1.5 × 10^7^ Gy was reported in thin films of dry poly(methyl methacrylate) under cryo-conditions (Beetz and Jacobsen, 2002). Furthermore, the observed soft X-ray-radiation induced changes of the sample surface might be caused by local heating effects, reported even at low temperatures (Ponomarenko et al., 2011). From a practical point of view, the strong bleaching of both fluorophores employed in this study, already by comparatively low-dose X-ray exposure during searching for regions of interest, suggests to apply in-column fluorescence always before soft X-ray cryo-microscopy. In our experience, light cryo-microscopic data only rarely provided reliable information whether the target area would be suitable for X-ray tilt series acquisition. Taking this into account, the above-mentioned limitation in the order of experimental steps reduces the efficiency to find correlations between fluorescence and X-ray data. An alternative would be to scan the entire vitreous sample grid in advance of mounting it to the X-ray microscope, by external fluorescence cryo-microscopy (for a review, see Briegel et al., 2010), thus saving precious beam time. However, most of the apparatuses described are optimized for the use of round 3.05 mm standard electron microscopic grids (Rigort et al., 2010), and adaptation effort is needed to use them with IFR-1 or HZB-2 grids.

Our tomographic resolution estimates for the 25 nm zone plate objective of the HZB TXM in Berlin, determined half-pitch at a cut-off threshold of 0.3 with ∼25 nm as NLOO-2D near zero-tilts (Fig. 6A, dashed line) or, more globally, with ∼35 nm from NLOO-3D curves (Fig. 6C, thick solid line), are in the same range as reported before (Carrascosa et al., 2009, Schneider et al., 2010). It should be emphasized that these resolution figures can only be compared, especially to results obtained by different imaging techniques, if data are treated with the same method for resolution assessment, as exemplified for soft X-ray and electron cryo-microscopy of vaccinia virus (Carrascosa et al., 2009). In order to characterize and to quantify the position-dependent impairment of tomographic resolution by the reduced depth of focus of zone plate objectives with higher numerical apertures, the use of radiation-resistant three-dimensional model objects seems to be inevitable (Knöchel 2005). Nonetheless, such studies are very important as they could yield algorithms to correct partially coherent soft X-ray microscopic tilt or focus series for blurring by a narrow depth of focus, unleashing the full resolution power of highly-developed, nanostructured X-ray diffraction optics also in 3D.

## 5 Conclusions

We conclude that soft X-ray cryo-microscopy/tomography is now even more attractive for structural cell biologists as it provides high contrast three-dimensional structural information with nanometer resolution from well-preserved biological objects up to 12 μm thick which can be spatially correlated with specific information from fluorescence cryo-microscopy. That opens the path to study the role of specific proteins in thick cellular objects such as the nucleus in detail, without the need of elaborate thinning techniques like serial sectioning. As shown, more information about the function of membranous structures like the nucleoplasmic reticulum – a novelty in cell biology that has been hard to access up to now – can thus be obtained.

## Conflict of interest statement

The authors declare no competing financial interests.

## Appendix A Supplementary data

Supplementary video S1 Dynamic superimposition of soft X-ray control images before (Fig. 3A) and after tilt series acquisition (Fig. 3B).

Supplementary video S2 Prealigned tilt series corresponding to data shown in Fig. 3C.

Supplementary video S3 Subtomogram corresponding to data shown in Fig. 4B.

Supplementary video S4 Negative contrast volume-rendered tomographic reconstruction corresponding to data shown in Fig. 4C.

Supplementary video S5 Dynamic superimposition of soft X-ray (Fig. 4C) and fluorescence (Fig. 4D) cryo-microscopic data.
