# Supplementary figures and images for: Correlative VIS-fluorescence and soft X-ray cryo-microscopy/tomography of adherent cells
Source: J Struct Biol. 2012 Feb;177(2-2):193–201. doi: 10.1016/j.jsb.2011.12.012 (PMC3343273; doi:10.1016/j.jsb.2011.12.012)

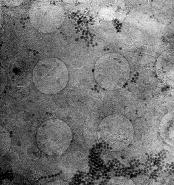

Supplement: Supplementary video S1 — Dynamic superimposition of soft X-ray control images before (Fig. 3A) and after tilt series acquisition (Fig. 3B). [file mmc1.jpg]

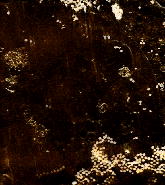

Supplement: Supplementary video S5 — Dynamic superimposition of soft X-ray (Fig. 4C) and fluorescence (Fig. 4D) cryo-microscopic data. [file mmc5.jpg]
